# Epigenetic Crosstalk between Malignant Plasma Cells and the Tumour Microenvironment in Multiple Myeloma

**DOI:** 10.3390/cancers14112597

**Published:** 2022-05-24

**Authors:** Alessandro Allegra, Marco Casciaro, Paola Barone, Caterina Musolino, Sebastiano Gangemi

**Affiliations:** 1 Division of Hematology, Department of Human Pathology in Adulthood and Childhood “Gaetano Barresi”, University of Messina, 98125 Messina, Italy; baronepaola2903@gmail.com (P.B.); cmusolino@unime.it (C.M.); 2 Unit of Allergy and Clinical Immunology, Department of Clinical and Experimental Medicine, School of Allergy and Clinical Immunology, University of Messina, 98125 Messina, Italy; marco.casciaro@unime.it (M.C.); gangemis@unime.it (S.G.)

**Keywords:** multiple myeloma, epigenetics, tumour microenvironment, chemoresistance, bone disease, angiogenesis, hypoxia

## Abstract

**Simple Summary:**

Multiple myeloma is a haematological malignancy due to a proliferation of clonal plasma cells. The medullary milieu consists of a bone marrow microenvironment comprising an assortment of cells and structures supporting blood cell production in the bone marrow. Epigenetic alterations can modify the interplay between multiple myeloma cells and the bone marrow niche. The continuous bidirectional relationship between bone marrow microenvironment cells and neoplastic plasma cells can be altered, profoundly modifying the natural history of myeloma. Our review focuses on the role of epigenetic mechanisms in the development and growth of multiple myeloma. It discusses the crosstalk between the tumour microenvironment’s epigenetic mechanisms, the progression of multiple myeloma, and the onset of multiple myeloma complications, such as bone disease.

**Abstract:**

In multiple myeloma, cells of the bone marrow microenvironment have a relevant responsibility in promoting the growth, survival, and drug resistance of multiple myeloma plasma cells. In addition to the well-recognized role of genetic lesions, microenvironmental cells also present deregulated epigenetic systems. However, the effect of epigenetic changes in reshaping the tumour microenvironment is still not well identified. An assortment of epigenetic regulators, comprising histone methyltransferases, histone acetyltransferases, and lysine demethylases, are altered in bone marrow microenvironmental cells in multiple myeloma subjects participating in disease progression and prognosis. Aberrant epigenetics affect numerous processes correlated with the tumour microenvironment, such as angiogenesis, bone homeostasis, and extracellular matrix remodelling. This review focuses on the interplay between epigenetic alterations of the tumour milieu and neoplastic cells, trying to decipher the crosstalk between these cells. We also evaluate the possibility of intervening specifically in modified signalling or counterbalancing epigenetic mechanisms.

## 1. Introduction

For a long time, the bone marrow microenvironment (BMME) was believed to be only a useful support, offering structure for the much more relevant clonal cells’ activity confined within. In recent years, however, the BMME has been identified as a composite tissue that, in addition to critical homeostatic actions in haematopoiesis, can stimulate haematological malignancies [1]. Furthermore, several findings suggest that neoplastic cells can alter the local BMME to promote their proliferation, to the detriment of non-malignant cells [1].

Multiple myeloma (MM) is a haematological malignancy caused by accumulated clonal plasma cells in the bone marrow (BM). Despite the insertion of numerous new therapeutic substances comprising oral proteasome inhibitors, immunomodulatory agents, and antibodies [2,3,4,5,6,7], MM is a generally incurable disease.

MM plasma cells gather in the BMME, where they contact BM stromal cells and stick to the extracellular matrix (ECM) and proteins [8]. These strict connections of the neoplastic plasma cells with their milieus are regulated by different factors, such as growth factors, adhesion molecules, and signalling elements [9]. Successive alterations of these factors are associated with acquired resistance to programmed cell death and augment cytokine transcription and delivery in BMME, which cause both the proliferation and survival of MM cells [8,9].

The present interpretation is that MM onset and evolution are determined by genetic mutations existing at the beginning of the disease or developed during the condition [10,11]. However, besides the well-known genetic alterations, several studies have now indicated that epigenetic modifications, such as abnormal DNA methylation [11,12,13] and histone change [14,15], and abnormal microRNA (miRNA) production [16,17], should be judged as relevant in influencing the pathogenesis of MM [18,19,20]. Epigenetic modifying substances, such as DNA methyltransferase or histone deacetylase inhibitors, are already being evaluated as possible agents for the therapy of MM [21,22].

### 1.1. General Consideration on Epigenetics

Epigenetics is the analysis of heritable molecular factors that are independent of phenotypic features, and different epigenetic modifications are possible. Chromatin is composed of DNA twisted around the nucleosome constituted of four histones. The most frequent epigenetic variations are histone changes and DNA methylation involving the fifth carbon atom of cytosine residues. These modifications cause a dense chromatin configuration, provoking transcriptional gene silencing. Contrariwise, DNA demethylation generates the onset of a free chromatin configuration and stimulates gene expression. Besides these variations, epigenetic control also comprises the control of three-dimensional chromatin status, dynamic location of nucleosomes, modification of non-coding RNA, and enhancer RNA [23].

Epigenetics modifications are now an essential element in tumour onset and diffusion. Moreover, these changes display unique characteristics in diverse cancer cells. These alterations’ specific profile is indicated as the epigenome, an essential determining factor of gene function and cell destiny [24].

### 1.2. Epigenetics, Bone Marrow Microenvironment, and MGUS

MM clonal plasma cells need sustenance from the BMME for survival [25,26]. In fact, in BM, we can identify a niche containing microenvironmental cellular elements, such as stromal cells, osteoclasts, osteoblasts, adipocytes, endothelial cells, T cells, and natural killer cells, which can modify the fate of neoplastic cells. Moreover, in the niche, other factors, such as the extracellular matrix and adhesion molecules (very late antigen-4, syndecan-1, and Vascular cell adhesion protein 1), are present to intervene in MM pathogenesis.

There are three different modalities of how the niche’s content stimulates the proliferation and survival of MM plasma cells: the discharge of soluble factors, direct cell–cell contact, and the liberation of extracellular exosomes [27,28]. However, epigenetic modifications can alter these activities. The constant bidirectional correlation between BMME cells and clonal plasma cells can be changed, intensely altering the natural history of myeloma, starting from the same progression from monoclonal gammopathy of uncertain significance (MGUS) to MM.

For instance, histone demethylation is a process in which the demethylation of lysine residues occurs through lysine demethylases (KDMs). The diminished production of the H3K4 demethylase KDM1A owing to a germline mutation in MGUS was reported to increase cell growth via *MYC* stimulation. Remarkably, transcriptomes from subjects with *KDM1A* alterations displayed enrichment in the pathways correlated with MM pathogenesis and MM BMME interactions versus *KDM1A* wild-type subjects, again indicating the relevance of epigenetic modifications in the relations between MM plasma cells and the BMME [29].

Contrary to what was noted with *KDM1A*, a different component of the lysine-specific demethylases, KDM6B, was reported to be augmented in MM. KDM6B is a demethylase of H3K27 modulated by NF-kB signalling [30]. *KDM6B* knockdown was observed to reduce MM cell proliferation. In contrast, TNF-α and culture media before being conditioned with MM bone marrow stromal cells (BMSC) could induce KDM6B and increase cell proliferation.

In our review, we will try to analyse whether the epigenetic changes that occur in MM patients may play a role in the onset of the disease and discuss the crosstalk between the epigenetic mechanisms of the tumour microenvironment and the progression of MM and the beginning of MM complications, such as bone disease. In our discussion, we have deliberately left out the alterations of the cells of the BBME due to non-epigenetic modifications. We have also limited the discussion of possible therapeutic interventions that target BMME cells, excluding interventions not aimed at modifying functional alterations not dependent on epigenetic alterations.

## 2. Bone Marrow Microenvironment and Multiple Myeloma

Cancer cells do not proliferate when separated from their surroundings, but they significantly institute solid links with the microenvironment essential for cancer advancement [31,32]. In contrast to solid tumours, where the positions of primary disease and secondary metastases are characteristically different, in MM, we have wide tumour diffusion in multiple places within the BM. The BM niche, thus, attains significance as a pathogenic element in MM, and the BM milieu has been reported to stimulate plasma cell growth and tumour cell transferral and homing [33]. It has been well ascertained that a bidirectional communicating circle is present between MM plasma cells and BMME cells. The BM milieu of MM patients varies in its cellular and noncellular arrangement from that of normal subjects [34].

MM plasma cells tend to be located in contiguity to osteoblasts (OBs) and the vascular endothelium, appropriating the BM niche [35,36]. However, stromal cells, adipocytes, macrophages, fibroblasts, osteoclasts (OCs), dendritic cells (DCs), and lymphocytes, modified in their functional activity, are other essential actors in MM pathogenesis [37].

### Epigenetic Regulation of the Bone Formation

Bone development is a procedure in which bone maturation is rigidly regulated by genetic factors and signalling pathways that is able to cause variations of the extracellular matrix and the formation of bone. Several of these genetic factors have been recognized, but it has become clear that the collaboration of genetic components with epigenetic factors is essential to regulate bone formation. Several epigenetic regulators acknowledged to participate in bone cell fate have been identified [38].

Osteoblasts are cells originating from mesenchymal stem cells that contribute to bone formation. Numerous genes and pathways can be subject to epigenetic regulation. For instance, the expression of runt-related transcription factor 2 (RUNX2) is essential for osteoblast maturation and is implicated in differentiation. In particular, RUNX2 functions as a primary transcription factor and is also involved in the osteogenic lineage’s growth, mobility, and commitment. Furthermore, it has been demonstrated that several pathways, such as bone morphogenetic protein, the Wnt/Notch system, and hedgehog signalling, are upstream of RUNX2 [39].

Osteoclasts are multinucleated cells that descend from the hematopoietic precursor of the myeloid line and can cause bone resorption. Their differentiation needs epigenetic control of gene expressions implicating chromatin dynamics. Recognising epigenetic controllers in osteoclasts has become the main objective of understanding the effects of these cells on clonal plasma cells and their role in multiple myeloma bone disease. The epigenetic changes considered in this review comprise DNA methylation, histone alterations, and noncoding RNAs.

## 3. Epigenetic Changes in Mesenchymal Stem Cells and Multiple Myeloma

Mesenchymal stem cells (MSCs) are cells that maintain the ability to self-renovate and differentiate into various cell types, comprising adipocytes, fibroblasts, chondrocytes, and OBs/osteocytes [40,41].

BM MSCs’ survival and maturation have been demonstrated to be due, in part, to the effects of different transferases and associated demethylases through the control of specific lineage-associated transcription factors [42,43,44,45,46,47,48].

### 3.1. Epigenetic Modifications and Maturation of Osteoblasts

Prior studies stated that MSCs from MM subjects are cytogenetically regular [49,50] but present changes in their proteomic or transcriptional configurations, even without MM cell contact [51]. This indicates that epigenetic modifications could be directing the tumour-stimulating activities of MSCs in MM. Experimentation displayed anomalous recruitment of chromatin remodellers in MSCs from MM subjects, participating in the transcriptional inhibition of Runx2, the primary regulator of OB differentiation [52].

Moreover, extensive DNA methylation changes of BM-isolated MSCs from different MM stages were recognized, especially in Homeobox genes implicated in osteogenic differentiation. Furthermore, these alterations are repeated in vitro by the contact of normal MSCs with MM cells. Pharmacological aiming of DNA DNMTs and G9a with the double inhibitor CM-272 restores the presence of osteogenic controllers and stimulates osteoblast differentiation of MM MSCs. Most significantly, using CM-272 avoids tumour-correlated bone damage and diminishes tumour mass in animal MM experimental models. These findings make evident that epigenetic alterations cause the diminishing of bone development. The use of the double inhibitor can overturn myeloma bone disease (MBD) [53].

Interestingly, as mentioned above, several Homeobox family genes are precociously altered in MM MSCs, and these genes are recognized as critical regulators of OB maturation. Their production is controlled by the demethylation of their promoters all through the osteogenic process. Furthermore, da Silva et al. confirmed that normal MSCs exposed to MM cells, similar to that described in patient MSCs, showed an abnormal methylome and presented altered MSC-to-OB differentiation. They also reported that these epigenetic alterations in MSCs happen even without direct contact with MM plasma cells, indicating the involvement of secretory processes. Thus, the alteration of osteogenesis in different phases of MM results from the early transcriptional alteration of Homeobox genes, and abnormal DNA methylation may be the central mediator in this mechanism [54].

Other epigenetic changes in the mesenchymal cells could be essential in MBD onset in MM subjects. Experimentation displayed that the blocking of bone formation is principally due to suppressing *RUNX2*, as RUNX2/CBFA1 is needed for OB maturation [55], and its production is decreased in precursors from MM subjects with lytic alterations [56,57].

**Figure 1:**
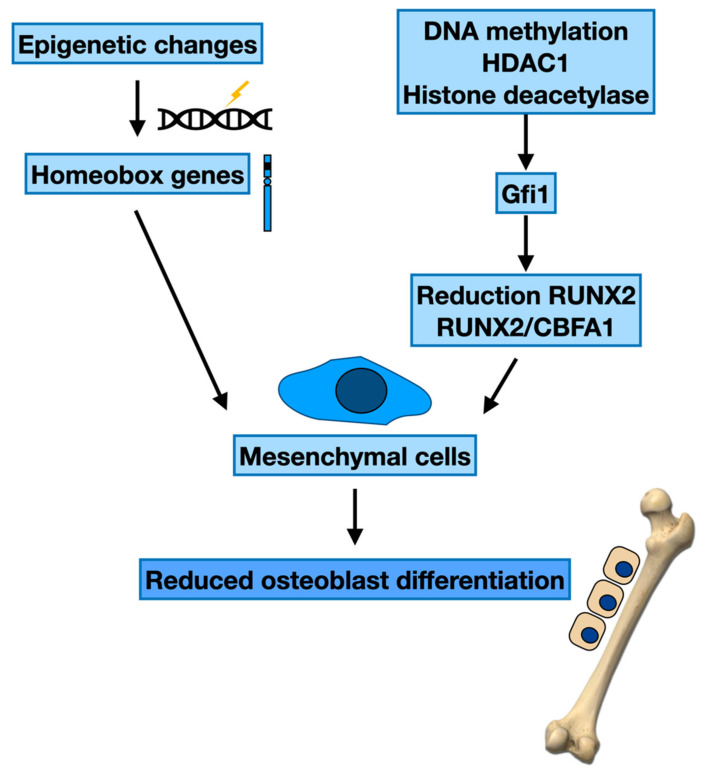
Epigenetic modifications and maturation of osteoblasts.

Other studies have evaluated the participation of the epigenetic changes of these systems in MM. Epigenetics-based mechanism experimentations in MM-BMSCs evidenced the effect of the transcription factor growth factor independence-1 (Gfi1) in inhibiting RUNX2 gene expression [58]. Gfi1 is a SNAG (Snail/Gfi1) domain including the C2H2 zinc finger implicated in the differentiation of lymphoid and myeloid cells [59], and novel studies have proposed its alterations in several hematologic tumours comprising MM [60,61,62,63]. BMSCs in contact with MM cocultures or gathered from a murine MM model or MM subjects have augmented Gfi1 expression. In addition, BMSC from Gfi1-knockout animals or *Gfi1* knockdown in murine OB precursors (pre-OBs) before MM exposition extremely safeguarded the cells from MM suppression with increased reaction to OB differentiation signals [58]. Significantly, knockdown of Gfi1 after MM contact of murine pre-OB or in patient-originated MM-BMSCs could annul the OB inhibition and augment the reaction to OB differentiation signals. Transcriptional inhibition by Gfi1 is determined by its recruitment of the histone-modifying enzymes HDAC1, methyltransferase G9a, and EZH2, and lysine-specific histone demethylase 1 (LSD1/KDM1A) to affect gene promoters [64,65]. Proof of Gfi1-mediated chromatin’s suppression of RUNX2 in the ambit of myeloma suppression derived from the demonstration that the boost of Gfi1 in preOBs blocked RUNX2 reporter expression. This was averted by employing the HDAC inhibitor (HDACi) Trichostatin A [58]. Different studies have analysed Gfi1 binding sites within the RUNX2 promoter and stated that, after MM contact, Gfi1 engages LSD1, EZH2, and HDAC1 to modify the bivalent signature of the RUNX2 promoter into one mainly methylated at H3K27me3. This inhibited heterochromatic condition at the RUNX2 promoter continued for numerous days after eliminating MM cells from the cocultures and was refractory to OB differentiation signals (Figure 1).

### 3.2. Epigenetic Changes and Osteoclast Differentiation

**Figure 2:**
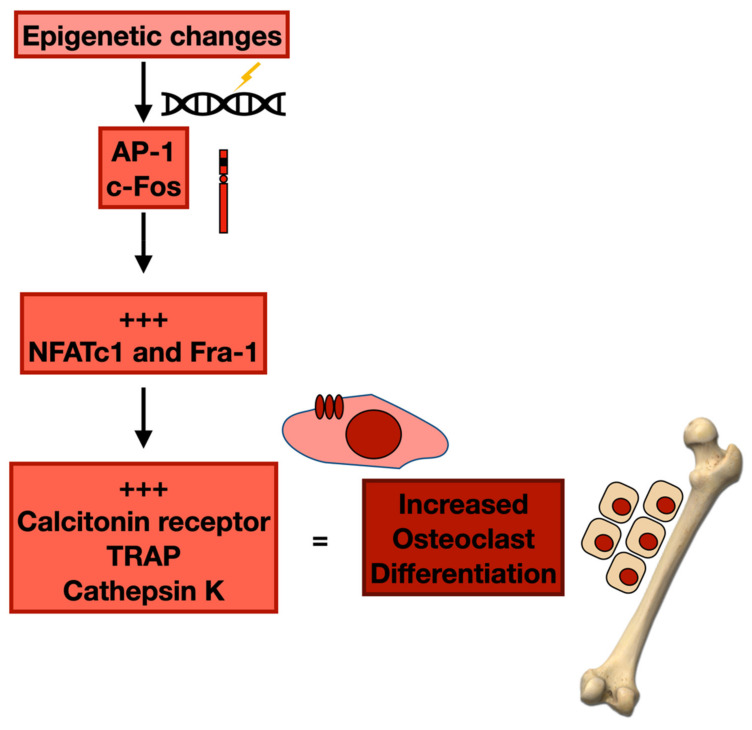
Epigenetic changes and osteoclast differentiation.

Several reports were also conducted on the RAF-MEK1/2-ERK1/2 cascade and downstream c-Jun/Fos and Activator protein-1 (AP1) pathways, which regulate numerous systems. Epigenetic changes stimulate or inhibit AP-1 functions and give the possibility to aim at AP-1 transcriptomes [66] selectively. In addition to c-Maf and MafB, whose amounts are associated with a less important MBD, other AP-1 components have been correlated with lytic bone lesions in MM [67,68]. Precisely, c-Fos works as a crucial transcription factor (TF) for OC differentiation. The absence of c-Fos determines the inhibition of OC differentiation and an augmented quantity of BM macrophages [69]. As far the mechanism, c-Fos expression is stimulated by Receptor Activator of NFκB Ligand (RANKL) and Macrophage Colony-Stimulating Factor (M-CSF), and augments the expression of Nuclear Factor of Activated T cells c1 (NFATc1) and Fra-1. In the last phase of OC differentiation, NFATc1 collaborates with c-Fos to stimulate OC-specific genes, such as *calcitonin receptor*, *TRAP*, and *cathepsin K* [70,71,72]. Furthermore, the number and persistence of OCs are regulated by Fra-2 via the effect of hypoxia and Leukaemia Inhibitory Factor (LIF). Fra-2 transcriptionally stimulates LIF through Fra-2/c-Jun heterodimers and regulates LIF/LIF-receptor/PHD2/HIF1α signalling. Moreover, Fra-2 transgenic animals display osteosclerosis with augmented bone development, while the bones of Fra-2-deficient animals have increased dimensions and amounts of OCs. Furthermore, Fra-2 controls OB differentiation via the transcriptional regulation of collagen1α2 and osteocalcin. In vivo, Fra-2-overexpressing animals are osteosclerotic. Analogously, a different component of Fos proteins, Fra-1, controls the function of OBs through the generation of bone matrix elements, such as collagen1 α2, osteocalcin, and matrix Gla protein. Animals with increased expression of Fra-1 develop osteosclerosis [73,74] (Figure 2).

MM plasma cells inhibit OBs via the production of the Wnt antagonist sclerostin, an osteocyte-produced negative controller of bone generation. Therefore, an anti-sclerostin antibody overruled the reduction of Fra-1, Fra-2, and c-Jun in BM stromal cells cultured with MM cells in an OB-differentiating medium [75]. HDACis, comprising trichostatin A (TSA), valproic acid (VPA), vorinostat (SAHA), and LBH589, inhibit the transcription of both Fra-1 and c-Jun, and so decrease c-Jun: Fra-1 heterodimer production [76], which may justify their anti-MM efficacy. For these reasons, HDACis have been accepted by the US FDA for MM therapy in subjects who have undergone two prior treatments [77].

Furthermore, protein arginine methyltransferases (PRMTs) can regulate arginine methylation in histone proteins and are implicated in abnormal epigenetic systems in tumours. The PRMT1 inhibitor TC-E 5003 (TC-E) reduces the nuclear transfer of c-Jun, as well as of NFκB subunits p65 and p50 and controls c-Jun expression after lipopolysaccharides administration [78]. Employing Selective Microfluidics-based Ligand Enrichment (SMiLE-seq), a new technique to evaluate protein-DNA interactions, resulted in a de novo motif discovery on all Jun: Fos heterodimers. It thus offered new understandings of partner-specific heterodimer DNA-binding preferences [79]. Furthermore, c-Jun: c-Fos combines with the DNA sequence, overturning epigenetic silencing [79,80]. Contrariwise, the anti-MM effect of molecules such as 5-azacytidine may be justified by the blockade of c-Jun: c-Fos binding [81] (Table 1).

The Ten-eleven translocation (TET) system represents a different regulatory system able to interfere in the bone disease of MM via epigenetic mutations. Nevertheless, this system still does not seem to have been well analysed in MM. The TET protein family is composed of two elements; TET1 is principally expressed by embryonic stem cells (ESC), while TET2 and TET3 are present in diverse cell lineages [82,83]. The evaluation of TET1 and TET2 knockout animals in the mesenchyme lineage demonstrated altered bone-generating ability in BMSC [84]. The global knockdown of all three TET substances recognized 1072 decreased genes and 729 augmented genes, indicating that TET proteins can stimulate or inhibit transcription [85]. Moreover, reprogramming fibroblasts into induced pluripotent stem cells (iPSC) caused increased amounts of TET1 and TET2 and a reduction of TET3 [86].

Cakouros et al. employed the siRNA knockdown of TET molecules and ascertained TET1 to be an inhibitor of osteogenesis [87]. TET1 stimulated the co-repressor agents, histone lysine methyltransferase, SIN3A, and EZH2 to osteogenic genes. On the contrary, TET2 was reported to be a stimulant factor of osteogenesis. The results proved that TET2 was responsible for the 5-hydroxymethylcytosine (5hmC—a regulator of DNA demethylation) levels on osteogenic lineage-associated genes, while TET1 also had an effect on this process. Remarkably, TET3 demonstrated no functional actions in BMSC osteo-differentiation.

**Figure 3:**
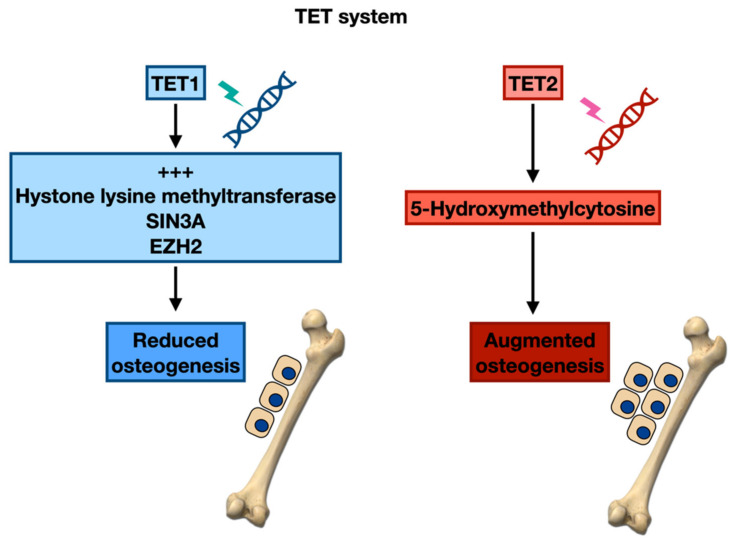
TET system’s influence on osteogenesis.

Furthermore, in an experimental animal model of ovariectomy-caused osteoporosis, clonogenic BMSC was considerably reduced, corresponding to the minor trabecular bone volume and decreased quantities of TET1, TET2, and 5hmC [87]. This report proved the epigenetic mechanism mediated via modifications in the DNA hydroxymethylation status controlling the stimulation of essential genes implicated in the lineage determination of skeletal stem cells. Affecting the TET components may present novel therapeutic approaches to avoid bone loss and lytic lesions in MM patients (Figure 3).

Finally, the family of proteins containing the bromodomain and extra-terminal domain (BET) motif attaches acetylated lysines to histones and engages molecules able to modify gene expression. The BET inhibitor JQ1 displayed powerful anti-growth functions on MM cell lines and reduced the MM tumour load in vivo [88].

However, BET repression reduced bone development in an experimental animal model [89]. Bromodomain 4 (BRD4) augments the production of OB-specific enhancers through the process of OB differentiation. Thus, alterations of BRD4 activity inhibit OB differentiation [90]. JQ1, a BET inhibitor, was employed to treat primary osteosarcoma [91]. By inhibiting c-MYC and RUNX2 expression, JQ1 decreased OB differentiation and tumour progression, suppressing c-MYC and RUNX2 expression [91,92]. Together, these findings propose that BET inhibitors may be useful in curing osteoblastic cancers. Still, their use in MM bone disease may be restrained due to their detrimental actions on osteogenic maturation, which could inhibit the recovery of bone alterations.

From what has been said so far, the role played by the epigenetic mutations of the cells of the medullary microenvironment in the onset and progression of bone disease in MM is evident. Epigenetic changes can provoke the inhibition of the osteogenic commitment of bone marrow cells. Furthermore, alterations of chromatin-modifying enzymes cause a transcriptional suppression of osteogenic genes in the BM milieu. Finally, further studies will confirm the possibility of using epigenetic inhibitors as valuable agents in the prevention or treatment of lytic lesions in MM.

### 3.3. Mesenchymal Stem Cells and Epigenetics: The Role of Exosomes and Non-Coding RNAs

MSCs from MM subjects have been reported to release exosomes that can enter MM plasma cells. In an experimental animal model, these exosomes derived from stromal cells stimulated plasma cell proliferation and MM progression via the transport of microRNAs (miRNAs), mitochondria, and proteins present in the exosomes [93].

MiRNAs are short non-coding RNAs implicated in the control of gene expression, and specific sets of miRNAs are diversely expressed in different forms of tumours [94,95,96,97,98].

While exosomes released by MM cells can modify the evolution of the disease and can intervene in the progression of the disease [99], exosomes originating from stromal cells derived from healthy subjects decreased MM plasma cell proliferation. Exosomes discharged by MMMSCs had minor concentrations of the MM suppressor miRNAs miR15a and miR161 than those originated from a healthy subject-MSCs [93]. Generally, the in vitro contact of MM plasma cells with exosomes originating from MM cells stimulated MM plasma cell growth, augmented the generation of IL-6, and increased the adhesion to fibronectin. Furthermore, exosomes originating from normal subjects showed anti-myeloma action in vitro and in vivo. A study reported that MM-MSCs presented augmented concentrations of miR-135b, causing a reduction of SMAD family member 5 (SMAD5) expression, and altered ability of OB differentiation [100].

An analogous action could be performed by other forms of non-coding genetic material, such as long non-coding RNA, whose altered distribution is known in MM patients [101,102].

For instance, MALAT1 was reported to interrelate with the Sp1 transcription factor in MSCs and stimulate the transcription of the latent TGF-β-binding protein 3 (LTBP3), an essential controller of TGFβ protein family members [103]. MALAT1 knockdown decreased the expression of LTBP3, which was concurrent with the decreased TGF-β1 amounts in MSCs originating from MM patients [103].

MEG3 is a different lncRNA that has been reported to be implicated in bone formation in MM by stimulating osteogenic differentiation. MEG3 is reduced in MM MSCs versus healthy MSCs and is related to a decrease in several osteogenic markers, such as RUNX2, osteocalcin, and osterix [104]. The authors proved that MEG3 augments the transcription of bone morphogenetic protein 4 (BMP4) by interrupting SOX2 binding to the BMP4 promoter, thus avoiding BMP4 silencing [104]. The blocking of TGF-β signalling is a possible anti-MM approach, as it stimulates bone formation and inhibits MM cell proliferation [105]. The participation of MALAT1 and MEG3 in controlling the TGF-β signalling pathway emphasizes the leading role of epigenetics in MM.

Alongside the effects of the epigenetic mutations of the cells of the BMME, a profound alteration of the proliferative dynamics of the neoplastic plasma cells can be caused by the epigenetic alterations induced by particular states of the BMME itself, such as a condition of hypoxia and a condition of acidity of the bone marrow.

## 4. Hypoxia and Epigenetic Alterations in Bone Marrow Microenvironment and Neoplastic Plasma Cells

It is well-known that the BM in MM is hypoxic and that reduced oxygen levels stimulate MM cell diffusion and angiogenesis [106]. Several results suggest that hypoxia modulates miRNA expression in MM cells [107,108]. For instance, an experiment established that hypoxic BM intensely reduces the expression of miR-199a-5p. MiR-199a-5p affects the transcription factor hypoxia-inducible factor-1α (HIF-1α), which is extensively expressed in MM plasma cells [109,110,111]. A study performed in hypoxic MM plasma cells demonstrated that using miR-199a-5p synthetic mimics decreased HIF-1α production and altered the motility of MM plasma cells and endothelial cells, augmenting the adhesion of neoplastic plasma cells to the hypoxic BMSCs. This result is entirely unexpected, as a different work suggested that hypoxia decreases MM cells’ sticking to the BM stroma [112]. In any case, miR-199a-5p synthetic oligonucleotides released in an experimental animal model of MM decreased plasma cell proliferation. They extended the overall survival of treated mice [113], indicating the anti-MM capacity of the miR-199a-5p replacement approach in affecting the hypoxic BM milieu in vivo [114].

Recently, a new oxidative epigenetic substance, RRx-001, with a specific mechanism of action versus azacitidine or decitabine, was discovered [115,116,117]. This molecule can modify haemoglobin and, in hypoxic situations, allows the transformation of nitrite to nitric oxide (NO), which gathers in inadequately oxygenated neoplastic tissues. NO quickly unites with superoxide in the hypoxic milieu to produce outstanding amounts of peroxynitrite, thereby inducing oxidative stress. Consequently, Rx-001 causes redox stress on neoplastic tissue, suppresses DNMTs and hypermethylation, and re-establishes tumour suppressor gene activity.

Das et al. stated that RRx-001 reduced the survival of MM cell lines and overwhelmed drug resistance [118]. However, the most exciting aspect is that RRx-001 reduced MM cell proliferation only in the presence of BM stromal cells. RRx-001 caused several effects, such as the induction of programmed cell death via the activation of caspases, generation of DNA damage through ATM/γ-H2AX, the liberation of ROS, and nitrogen species, and decreases in DNMT and global methylation. Studies employing RNA interference demonstrated an overall effect of DNMT1 in MM cell viability versus DNMT3a or DNMT3b. Deubiquitylating enzyme USP7 augmented DNMT1 functioning, while USP7-siRNA decreased DNMT1 function and reduced MM cell survival.

**Figure 4:**
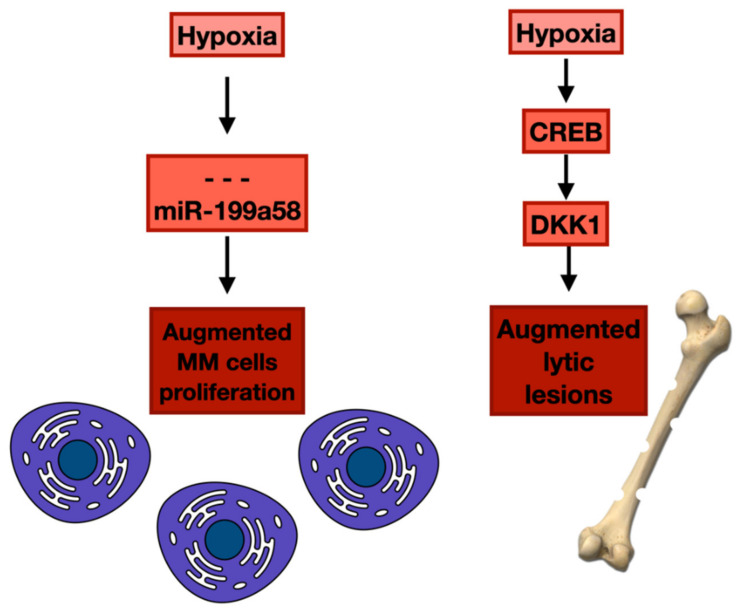
Hypoxia’s influence on MM cells’ proliferation and lytic lesions development.

Furthermore, RRx-001 plus USP7 inhibitor P5091 presented a synergistic effect against MM plasma cells. Regarding in vivo studies, MM xenograft models demonstrated that RRx-001 is safe, blocked MM cell proliferation, and prolonged survival. Joining RRx-001 with bortezomib or pomalidomide also caused a synergistic effect [118]. These findings offer a basis for the translation of RRx-001 to clinical use in MM patients (Figure 4).

Investigations have demonstrated that BM hypoxia might be involved in the genesis of bone lesions in MM patients. Numerous factors have been recognized to block osteoblast formation, comprising dickkopf-1 (DKK1). Different experiments have displayed that DKK1 can inhibit osteoblasts and augment the function of osteoclasts, provoking an alteration in bone metabolism [119].

A study supported that hypoxia stimulates DKK1 production in myeloma cells. In hypoxic situations, cAMP-responsive element-binding protein (CREB) activated DKK1 transcription. Furthermore, great amounts of DKK1 were correlated with the onset of lithic lesions in subjects with t(4; 14) MM. These patients presented an augment of the histone methyltransferase MMSET, which was recognized as a target gene of HIF-1α. In addition, CREB engaged MMSET, provoking the steadying of HIF-1α protein and the augmented methylation of histone H3 on the DKK1 promoter. Silencing CREB reduced the inhibition of osteoblast formations by myeloma-produced DKK1. The simultaneous administration of a CREB inhibitor and the hypoxia-activated prodrug TH-302 decreased bone disease [120].

In conclusion, it is relevant to note that anti-apoptotic ability may be developed under both normoxic and hypoxic situations. Still, the mechanisms are different, as they are regulated by factors such as IRF4 and MYC under normoxic conditions and by HIF in hypoxic cases. HIF, which may be stimulated by epigenetic changes in the BM microenvironment, intensely augments the transcription of different genes implicated in glycolysis and angiogenesis, provoking chemo-resistance, and cell proliferation. MM cell survival may be due to the switching of their regulatory factors from IRF4 and MYC to HIF. This suggests that, to attain long-lasting remission, a treatment effective against HIF may be a successful therapeutic approach for MM.

## 5. Epigenetic Changes in the Bone Marrow Microenvironment and Chemoresistance in Multiple Myeloma

Multiple myeloma cell relations with the BM milieu influence acquired drug resistance and disease relapse. Abnormal gene methylation might be essential in the onset of these phenomena.

MM cells attain resistance to anti-MM drugs via connections with the BM microenvironment through different systems. BMSCs generate soluble factors, such as insulin-like growth factor-1 and interleukin-6, to stimulate the signal transduction pathways causing soluble factor-mediated drug resistance. Furthermore, BMSCs augment the expression of ABC drug transporters, antiapoptotic members of the Bcl-2 family, and cell cycle inhibitors in myeloma cells after direct contact provoking the so-called cell adhesion-mediated drug resistance (CAM-DR). The clarification of the epigenetic mechanisms causing drug resistance may significantly influence the success of MM treatments.

Comparing the gene expression profiles of cell lines that acquired melphalan resistance through adhesion to fibronectin demonstrated that post-transcriptional systems principally cause CAM-DR, and the resistance is correlated with a precise transcriptome modification [121]. VLA-4-mediated CAM-DR is accompanied by G1 cell cycle blocking, a reduction in Cyclin A and Cyclin E function, and an augmentation of CDK inhibitors p21Cip1/Waf1 and p27Kip1. Moreover, this adhesion mechanism can provoke resistance to programmed cell death caused by Fas ligand in MM cells through post-transcriptional mechanisms, such as an augmented relocation of c-FLIP, which inhibits the death-inducing signalling complex [122].

The BMME can also cause the onset of drug resistance by altering other epigenetic factors, such as H3K27. MM cell contact with the BMME stimulates the phosphorylation of EZH2, which becomes inactive, and this phenomenon overturns drug-induced hypermethylation at H3K27. The demethylation of this factor provokes the stimulation of antiapoptotic genes, such as *IGF1*, *BCL2*, and *HIF1α*. These findings indicate that epigenetic treatment blocking the IGF-1R/PI3K/Akt pathway might be an encouraging method to overwhelm the treatment resistance by inducing EZH2 dephosphorylation and H3K27 hypermethylation [123].

The BMME can also influence chemoresistance by modulating miRNAs. The stroma-provoked reduction of miR-101-3p and the augmentation of survivin have been reported to defend MM cells against several treatments [124]. Modulating the miR-101-3p/survivin axis in MM by an increase in miR-101-3p or by the silencing of survivin causes programmed cell death, even in the presence of BMSCs, thus overwhelming the microenvironment-caused drug resistance [124].

### Epigenetic Effects of Proteasome Inhibitors

Proteasome inhibitors can disable CAM-DR by altering the VLA-4-caused contact of MM cells with BMSCs [125]. It was demonstrated that the reduction of this molecule caused by proteasome inhibitors is not due to an effect on NF-κB, but epigenetic mechanisms. Pharmacological blocking of HDACs can cause this process [126]. Proteasome inhibitors reduce the transcription of class I HDAC genes via caspase 8-mediated cleavage of Sp1 transcriptional activator [127,128]. Moreover, HDAC1, 2, and 3 modify the response of MM plasma cells to proteasome inhibitors. HDAC1 is highly present in CD138-negative MM cells and reduces the transcription of CHOP and Xbp1, which may induce the innate resistance of myeloma stem cells [129]. Thus, HDAC inhibitors could make sensitive myeloma stem cells to MM treatment by re-establishing the production of CHOP and Xbp1 [130]. This hypothesis might justify the synergistic action of bortezomib with panobinostat in the PANORAMA trials [131,132,133].

Exploiting the epigenetic capacities of drugs traditionally used in the treatment of MM or associating drugs capable of intervening in the epigenetic modifications of the cells of the medullary microenvironment could constitute a new approach for the therapy of MM and prevent the onset of resistance to chemotherapy.

As mentioned above, H3K27me3 was recognized as a critical histone modifier for CAM-DR in MM plasma cells [113]. Cell adhesion responds to anti-cancer drug-provoked hypermethylation of H3K27 through Akt-caused deactivating phosphorylation of EZH2, which supports the activation of anti-apoptotic genes, such as *IGF1*, *BCL2*, and *HIF-1 alpha*, to induce drug resistance in MM cells. The repression of the IGF-1R/PI3 K/Akt system could overturn CAM-DR by provoking EZH2 dephosphorylation and H3K27 hypermethylation in vitro in experimental animal models. Several molecules neutralizing EZH2 phosphorylation, such as CDK, PI3K/Akt, and IGF-1R inhibitors, were efficacious in contrasting CAM-DR in vitro and in vivo. Of particular interest is the action of the IGF-1R inhibitor OSI-906, which seems to be exceptionally efficient for overwhelming CAM-DR. This effect might be transferred to the clinic to enhance the response to therapy of MM subjects in combination with traditional anti-MM drugs [134].

## 6. Epigenetic Alterations of the Bone Marrow Microenvironment and Treatment of Multiple Myeloma

Recently, elaborating new therapeutic approaches that could operate on the neoplastic plasma cells and the BM milieu and their communications has aroused much interest. Numerous experiments indicate that HDACi have a relevant effect interfering with this axis. For instance, LAQ824 and panobinostat can destroy MM plasma cells, even when grown with stromal cells [135,136]. Furthermore, the gene expression profiling of alterations caused by vorinostat administration suggested that this molecule reduced transcripts for IL-6 receptor and IGF/IGF-1-R [137]. Further experimentation reported that vorinostat reduced the production of IL-6 by the BM stromal cells after contact with MM plasma cells [138].

A different mechanism must be considered in studying the relationships between epigenetic alterations, BM milieu and the onset of MBD, and the modification of angiogenic dynamics. In a study, the administration of JNJ-26481585 (an orally active pan-HDAC inhibitor) to 5T2MM-bearing animals caused a relevant reduction in angiogenesis and a vital slowdown in the onset of MBD [139]. Similarly, panobinostat inhibited the trabecular bone density reduction in in vivo studies [140]. A different molecule that has an antiangiogenetic effect via the reduction of vascular endothelial cell growth factor (VEGF) is VPA [141]. The administration of this substance reduced the vascular tubule generation caused by the coculture of MM plasma cells with osteoclasts [142] and reduced osteoclast-provoked MM cell growth and osteoclastogenesis [142]. VPA acid or TSA administration to mesenchymal stem cells augmented preferentially osteogenic differentiation with respect to other types of differentiation [143,144]. An experiment demonstrated that HDACi stimulated the expression of *RUNX2* and other osteogenic genes and displayed dose-related positive effects on OB differentiation [145]. Small-dose administration of HDACi augmented mineralized nodule generation by pre-OBs, although greater dosages of HDACi displayed significant cytotoxic actions [145].

More relevantly, combined administration of a low dose of the HDACi JNJ-26481585 with bortezomib provoked a more marked decrease in osteoclasts and augmented OBs, the trabecular bone size, and trabecular amount with respect to bortezomib employed as a single drug [146].

The repression of EZH2 has been correlated with osteo-protective effects and the EZH2i, GSK126, and augmented bone density in animal experimental models after bilateral ovariectomy [147,148,149]. The bone-protective action of EZH2i was also reported in a different study, which proved that the in vivo dispensation of 3-Deazaneplanocin A, a histone methyltransferase inhibitor, augmented the osteogenic differentiation of BMSCs [150].

These results clarify the composite molecular consequences of epigenetic modifiers on the MM microenvironment and postulate a justification for the therapeutical use of epigenetic drugs, alone or combined with traditional therapies, to improve prognosis in MM patients.

## 7. Conclusions

The BMME has a crucial effect on MM plasma cell growth and motility in the onset of chemoresistance and radioresistance [151]. The epigenetic heterogeneousness in MM participates in these effects and influences the possibility of relapse [152]. BMME and its cellular and humoral components are not adequately explored in routine practice. MM subjects are not differentiated by these elements, although it is probable to hypothesize that the effects of the BMME vary between the diverse patients. Growing suggestions show that BMME epigenetic alterations may modify MM progression and the occurrence of MM complications, such as osteolysis and chemoresistance. Targeting the epigenome to block the relationships between MM plasma cells and the BMME might still be a productive approach in the future. If the therapeutical objective is not a direct action on the MM plasma cells but, instead, an attempt to modify the BMME’s influence, the use of low dosages of epigenetic drugs combined with the dispensation of traditional anti-MM substances might be able to inhibit the microenvironment-caused pro-tumoural effects and decrease MM-correlated events with tolerable toxicities [153].

Finally, the epigenetics’ heterogeneity might clarify the variance in terms of treatment response. For instance, in a recent study, primary BM stromal cell culture supernatant reduced CD38 expression and decreased daratumumab-derived antibody-dependent cellular cytotoxicity [154].

In the future, it is possible that a better understanding of the epigenetic interactions in MM BMME may disclose new comprehension of MM pathogenesis, new disease markers, and hopefully the elaboration of new, personalized treatment approaches supporting more efficacious disease control.

## Figures and Tables

**Table 1:** Effects of epigenetic changes in the bone marrow microenvironment on MM progression and complications.

Cells	Epigenetics Changes	Genes	Target Cell	Effect	Ref.
MSC	DNA methylation	Homeobox genes	Osteoblast differentiation	MBD MM plasma cell growth	[53]
MCS	DNA methylation	Homeobox genes	Osteoblast differentiation	Effect on osteogenesis	[54]
MSC	Histone deacetylation	RUNX2 RUNX2/CBFA1	Osteoblast differentiation	Osteogenesis	[55,56,57]
MSC	Histone modifying enzymes	Gfi1	Osteoblast differentiation	Osteogenesis	[58]
MSC	Histone deacetylation	Fra-1, c-Jun	Osteoblast proliferation	MBD	[76,81]

MSC, mesenchymal stem cells; MBD, Myeloma bone disease; Gfi1, Growth Factor Independence-1; Fra-1 Fos-related antigen 1; RUNX2, runt related transcription factor 2.

## Data Availability

Not applicable.
